# Hepatitis B virus-associated hepatocellular carcinoma in South Africa in the era of HIV

**DOI:** 10.1186/s12876-020-01372-2

**Published:** 2020-07-13

**Authors:** Tongai Gibson Maponga, Richard H. Glashoff, Hannali Vermeulen, Barbara Robertson, Sean Burmeister, Marc Bernon, Jones Omoshoro-Jones, Paul Ruff, Alfred I. Neugut, Judith S. Jacobson, Wolfgang Preiser, Monique I. Andersson

**Affiliations:** 1 Division of Medical Virology, Stellenbosch University, Faculty of Medicine and Health Sciences, Cape Town, South Africa; 2 Division of Medical Microbiology & Immunology, Stellenbosch University, Faculty of Medicine and Health Sciences, Cape Town, South Africa; 3 Tygerberg Business Unit, National Health Laboratory Service, Cape Town, South Africa; 4 Division of Radiation Oncology, Stellenbosch University, Faculty of Medicine and Health Sciences, Cape Town, South Africa; 5 Division of Radiation Oncology, University of Cape Town, Cape Town, South Africa; 6 Department of Surgery, University of Cape Town, Cape Town, South Africa; 7 Department of Surgery, University of Witwatersrand, Johannesburg, South Africa; 8 Division of Medical Oncology, University of Witwatersrand, Johannesburg, South Africa; 9 Department of Medicine, College of Physicians and Surgeons, Columbia University, New York, USA; 10 Herbert Irving Comprehensive Cancer Center, College of Physicians and Surgeons, Columbia University, New York, USA; 11 Mailman School of Public Health, Columbia University, New York, USA; 12 Department of Microbiology, Oxford University Hospitals NHS Foundation Trust, Oxford, UK

**Keywords:** Hepatitis B infection, HIV infection, Hepatocellular carcinoma, Natural history, Age at presentation, Survival

## Abstract

**Background:**

Patients co-infected with hepatitis B virus (HBV) and the human immunodeficiency virus (HIV) are at risk of developing hepatocellular carcinoma (HCC). In sub-Saharan Africa, the overlap between high HIV and HBV prevalence may increase the incidence of HCC. This study investigated the impact of HBV/HIV co-infection on age at presentation and survival of HCC.

**Methods:**

Ethical approval was obtained to recruit, following informed written consent, patients diagnosed with HCC at oncology units at four South African hospitals. Between December 2012 and August 2015, patients newly diagnosed with HCC were recruited and provided demographic and clinical data and blood specimens. Patients were tested for HBV, hepatitis C virus (HCV) and HIV. Survival data was available for a subset of patients.

**Results:**

Of 107 HCC cases, 83 (78%) were male. Median age was 46 years (range 18 to 90 years), 68/106 (64%) were HBsAg-positive, and 22/100 (22%) were HIV infected. Among HBV surface antigen (HBsAg)-positive HCC cases, 18/66 (27%) were HIV-infected compared to 3/34 (9%) among those that were HBsAg-negative (*p* = 0.04). A greater proportion of HBV/HIV co-infected cases were female than HBV mono-infected (6/18, 33% vs 6/47, 13%; *p* = 0.005). In addition, HBV/HIV co-infected females presented at a younger mean age (36.8 years) than HBV mono-infected women (50.5 years) (*p* = 0.09). Median survival was 82 days among the HIV-infected HCC patients compared to 181 days among those without HIV (*p* = 0.15).

**Conclusions:**

HCC is an important complication in the HIV/HBV infected patient. HIV-positive patients presented with HCC at a younger age than HIV-negative patients, this effect appears to be greater in women. These data provide more evidence supporting the call to address. HCC as a cause of morbidity and mortality in the HBV/HIV co-infected patient population. (281 words).

## Background

Hepatocellular carcinoma (HCC) is the second most common cause of cancer mortality worldwide [1]. Sub-Saharan Africa (SSA) has the second highest number of HCC cases worldwide after South East Asia [2]. It is estimated that 60% of all HCC cases in developing countries are due to chronic HBV infection (CHB) [3]. This is despite the availability of a safe and effective vaccine for over three decades.

HCC in African populations appears to affect men more commonly than women [4]. Patients tend to present very late with malignancy and time to death post-diagnosis is short. The data from previous studies suggests that the average survival time from the onset of symptoms is around 11–12 weeks and 6–7 weeks from the time of diagnosis [5, 6], with less than 10% of patients surviving beyond a year after diagnosis [7].

CHB has an intermediate to high prevalence (5–7%) among adults in southern SSA [8], although studies suggest the prevalence in South Africa is lower than this at around 2–3% [9, 10]. SSA has the highest number of people living with human immunodeficiency virus (HIV) infection [11]. HIV as a risk factor for the development of cancer was first recognized because of its strong association with other virally driven malignancies, such as Kaposi’s sarcoma secondary to human herpes virus-8 (HHV8) infection, and non-Hodgkin’s lymphoma with Epstein-Barr virus (EBV) infection. These cancers usually develop in patients with untreated HIV infection [12, 13] and are now declining because of combined antiretroviral therapy (ART) [14]. However, the improved longevity in treated HIV infection has increased the incidence of other serious non-AIDS-related malignancies, including HCC [15–17].

Little is known about the natural history of HCC in the HBV/HIV co-infected patient. In fact, the question of whether HIV increases the risk of HCC in patients with CHB still remains largely unanswered. Two studies from resource rich settings suggest that HIV may hasten the evolution of HBV-related HCC, resulting in an earlier age of presentation in the HIV co-infected patient [18, 19]. These data were confirmed in a small West African study of 60 HCC patients in which seven HIV-infected cases presented at a younger median age of 32 years [interquartile range: 31–44] compared to 49 years [interquartile range; 44–59] among 53 patients without HIV infection [13]. A case-control study conducted within the Swiss HIV Cohort Study reported that decreased CD4+ cell counts were associated with an increased risk of HCC development among HIV-infected patients who were co-infected with HBV [20]. However, a recent case-control study in South Africa did not find an increased risk for HCC in HIV-infected individuals [21]. Little is known about HCC in the HIV/HBV infected African patient. The aim of this study was to describe the natural history of HCC in an HBV/HIV co-infected and monoinfected population.

## Methods and materials

### Study population

Between December 2012 and August 2015, patients who were newly diagnosed with primary HCC were prospectively recruited into the study from oncology departments at four university teaching hospitals in South Africa: Tygerberg Hospital and Groote Schuur Hospital in the Western Cape Province and Chris Hani-Baragwanath Academic Hospital and Charlotte Maxeke Johannesburg Academic Hospital in Gauteng Province. The diagnosis of HCC was based on radiological examination by contrast-enhanced computed tomography, abdominal ultrasound scan and/or magnetic resonance imaging and clinical findings. An experienced oncologist reviewed all patients. A subset of 25 cases also had histologic diagnosis of HCC. Serum alpha-fetoprotein (AFP) was measured, but levels < 400 μg/L did not exclude diagnosis of HCC. Following consent patients were interviewed using a standardized questionnaire, had a physical examination and provided blood samples. Clinical laboratory data were extracted from clinical records. A sub-group of the patients recruited from the Western Cape, underwent follow up for survival analysis. This was achieved by means of following up cases via the South African Department of Home Affairs to determine date of death (where applicable) using the national identification number.

### Ethical considerations

All participants were recruited following written informed consent according to the Declaration of Helsinki 2008. Ethical approval for the study was obtained from the health research ethics committees at Universities of Stellenbosch, Cape Town and Witwatersrand.

### Laboratory testing

Blood specimens were collected in EDTA anticoagulant and SST tubes with silica clot activator upon enrolment into the study. The tubes were centrifuged to separate plasma or serum and stored at − 80 °C until they were used for serologic and molecular testing. The HBsAg test was performed using the DiaSorin Murex HBsAg Version 3 immunoassay kit (DiaSorin, Saluggia, Italy). HBeAg and antibodies to HBeAg (anti-HBe) were tested using the ETI-EBK PLUS and ETI-AB-EBK PLUS (DiaSorin), respectively. Total antibodies against HBV core antigen (anti-HBc) were measured using the Murex Anti-HBc (total) immunoassay kit (DiaSorin). Antibodies to HCV and HIV were tested on the Abbott Architect i2000SR immunoanalyzer (Abbott Laboratories, Abbott Park, Illinois, USA). HBV DNA quantification and genotyping were performed as previously described on HBsAg-positive samples [22, 23]. HBV genotypes were obtained using the online genotyping databases of Stanford University, the Max Planck Institute for Informatics and the National Library of Medicine HBV genotyping tool. HCV viral load was tested using the COBAS AmpliPrep/COBAS TaqMan HCV assay.

### Statistical testing

All data entry and storage were performed in Epi-Info 7 (Centres for Disease Control, GA, USA) and statistical analysis was carried out in Statistica version 12 (StatSoft, Oklahoma, USA). Non-parametric data or small-sample data were described using medians and interquartile ranges and compared using the Mann-Whitney test where there were only two groups or the Kruskal-Wallis test in cases of three or more groups. Data with normal distribution were described using means and 95% confidence intervals. Comparisons were performed using the unpaired t-test where data were normally distributed. Categorical data were described using proportions and analysed using the chi-square test or Fisher’s exact test, depending on the number of observations. Survival after diagnosis of HCC was evaluated using the Kaplan-Meier method, and comparisons between patients infected and uninfected with HIV were performed with the log-rank test. Graphs were constructed using GraphPad Prism version 5 (GraphPad Software, CA, USA). All hypothesis testing was done at 95% confidence intervals and results were regarded as significant if *p* < 0.05.

## Results

### Demographic and clinical characteristics

A total of 107 HCC patients were recruited between December 2012 and August 2015. The mean age was 46 years (95% CI: 43–49), range 18–90 years. The majority were male 78% (83/107) and 55% (59/107) were Black African. Twenty two percent (22/100) of cases were HIV infected, of whom 91% (20/22) had evidence of current or past HBV infection. There was insufficient specimen for HIV testing for seven cases. Table 1 shows the demographic and clinical characteristics of the 100 patients with known HIV status.

**Table 1:** Demographic and clinical characteristics of HCC cases by known HIV status

	HIV-infected, N (%)	HIV-uninfected, N (%)	***p***-value
	22 (22)	78 (78)	
**Age groups (in years)**			
18–34	5 (23)	23 (29.5)	0.003
35–49	14 (64)	18 (23)	
50–59	2 (9)	23 (29.5)	
60–90	1 (5)	14 (18)	
**Gender**			
Male	14 (64)	64 (72)	0.03
Female	8 (36)	14 (18)	
**Race**			
African	20 (91)	32 (43)	0.0004
Caucasian	0 (0.0)	6 (8)	
Mixed	2 (9)	36 (49)	
*Missing data*^a^	*0*	*4*	
**Place of birth**			
Urban	14 (70)	44 (60)	0.4
Rural	6 (30)	29 (40)	
*Missing data*^a^	*2*	*5*	
**Alcohol dependence**			
Yes	5 (24)	15 (21)	0.7
No	16 (76)	57 (79)	
*Missing data*^a^	*1*	*6*	
**Known cirrhosis**			
Yes	5 (25)	15 (22)	0.8
No	15 (75)	52 (78)	
*Missing data*^a^	*2*	*11*	
**HBsAg status**			
Positive	18 (82)	47 (60)	0.06
Negative	4 (18)	31 (40)	
**Anti-HBc status**			
Positive	20 (91)	62 (83)	0.5
Negative	2 (9)	13 (17)	
*Missing data*^a^	*0*	*3*	
**Anti-HCV**			
Positive	1 (5)	7 (9)	0.7
Negative	20 (95)	68 (91)	
*Missing data*^a^	*1*	*3*	

Missing data^a^ not included in the statistical analysis

The median serum AFP was 6176 μg/L (interquartile range: 120–47,750). Of 98 cases, 69 (70%) had serum AFP greater than 400 μg/L. The majority of cases were diagnosed using imaging modalities in combination with clinical laboratory data. Contrast-enhanced computed tomography (CT) scan was done in 77 of 107. Liver histological examination was done on 31/100 of cases on whom information was provided (data was missing on seven cases).

### Prevalence of HBV serological markers

Of 106 serum samples tested (one had insufficient volume for testing), 68 (64.1%, 95% CI: 59–77) were seropositive for HBsAg, and 85/103 (82.5, 95% CI: 76–90) were anti-HBc positive. The mean age of the HBV-infected cases was 44 years (95% CI: 41–47) compared to 49 years (95% CI: 43–54) among those HBV-uninfected, *p* = 0.16. Among the 34 HBsAg negative samples, 17 (50%, 95% CI: 32–65) were positive for anti-HBc, indicating HBV exposure. Male HBsAg positive patients were younger (mean age 43.8 years, 95% CI: 40.1–47.5) than male HBsAg negative patients (mean age 50.4 years, 95% CI: 44.2–56.9), (*p* = 0.07). However, the HBsAg-positive female patients were similar in age (45.2 years, 95% CI: 35.8–54.6) to the HBsAg negative females (45.6 years, 95% CI: 32.9–58.2).

Of the 68 HBsAg seropositive patients, 65 were tested for HBeAg. There was insufficient serum in three cases. The overall prevalence of HBeAg among the HBsAg positive cases was 20/65 (31%). Five of 11 female patients (45%, 95% CI: 15–74) were HBeAg-positive, while only 15 of 54 male patients (28%, 95% CI: 14–40) were HBeAg positive, *p* = 0.29.

### Association of HBV/HIV co-infection with demographic and clinical characteristics at presentation of HCC

Table 2 shows the demographic and clinical characteristics of the 100 patients with known HIV status. Of the 22 HIV-infected patients, 14 were receiving cART. Nine were prescribed tenofovir, lamivudine and efavirenz; one was on lamivudine, stavudine and nevirapine; for the remaining four, no data was available. The median CD4+ T cell count at time of HCC diagnosis was 293 cells/μL (IQR: 200–602).

**Table 2:** Demographic and clinical characteristics according to known HIV and HBsAg status

	HIV+ HBV+	HIV- HBV+	HIV+ HBV-	HIV- HBV-	
	18 (18%)	47 (47%)	4 (4%)	31 (31%)	p-value
**Age groups (in years)**					
18–34	4 (22)	15 (32)	1 (25)	8 (26)	
35–49	12 (67)	13 (28)	2 (50)	5 (16)	0.04
50–59	1 (5.5)	11 (23)	1 (25)	12 (39)	
60–90	1 (5.5)	8 (17)	0 (0)	6 (19)	
**Gender**					
Male	12 (67)	41 (87)	2 (50)	23 (74)	0.1
Female	6 (33)	6 (13)	2 (50)	8 (26)	
**Ethnicity**					
Black African	16 (89)	23 (50)	4 (100)	9 (32)	0.006
Caucasian	0 (0)	3 (7)	0 (0)	3 (11)	
Mixed ancestry	2 (11)	20 (43)	0 (0)	16 (57)	
*Missing data*	*0*	*1*	*0*	*3*	
**Alcohol dependence**					
Yes	4 (22)	8 (18)	1 (3)	7 (25)	0.9
No	14 (78)	36 (82)	2 (67)	21 (75)	
*Missing data*	*0*	*3*	*1*	*0*	
**Known cirrhosis**					
Yes	5 (29)	12 (27)	0 (0)	3 (13)	0.4
No	12 (71)	32 (73)	3 (100)	20 (87)	
*Missing data*	*1*	*3*	*1*	*5*	
**HBeAg positive**					
Yes	10 (59)	9 (20)	n/a	n/a	0.003
No	7 (41)	37 (80)			
*Missing data*	*1*	*1*			
**HBV genotypes**					
A	9 (100)	25 (76)	n/a	n/a	0.00009
D	0 (0)	7 (21)			
E	0 (0)	1 (3)			
**AFP > 400 μg/L**					
Yes	11 (61)	35 (80)	3 (75)	14 (54)	0.1
No	7 (39)	9 (20)	1 (25)	12 (46)	
*Missing data*	*0*	*3*	*0*	*5*	
**Child-Pugh**					
A	9 (60)	30 (68)	2 (67)	14 (59)	1.0
B	5 (33)	11 (25)	1 (33)	8 (33)	
C	1 (7)	3 (7)	0 (0)	2 (8)	
*Missing data*	*3*	*3*	*1*	*7*	
**Nodules at diagnosis**					
Single lesion < 2 cm	3 (19)	5 (12)	0 (0)	4 (14)	
Single lesion 2–5 cm	0 (0)	0 (0)	0 (0)	1 (4)	1.0
Single lesion > 5 cm	2 (12)	6 (14)	0 (0)	4 (14)	
Multiple lesions	11 (69)	31 (74)	1 (100)	19 (68)	
*Missing data*	*2*	*5*	*1*	*3*	

Missing/unknown data, (including cases with unknown HIV status) are excluded from the table and statistical analysis

The mean age of HIV-infected patients at HCC diagnosis was 39.7 years (95% CI: 36.0–43.5); that of uninfected patients was 46.4 years (95% CI: 42.8–50.0), *p* = 0.07. The mean age of HIV-infected female patients was 35.8 years (95% CI: 34.1–40.0), 13 years younger than the mean age of HIV-uninfected females, 48.5 years (95% CI: 37.4–59.6), *p* = 0.08. The mean age of HIV-infected male HCC cases was 42.0 years (95% CI: 36.6–47.4), the mean age of HIV-uninfected males was 45.9 years (95% CI: 42.2–49.8) *p* = 0.4.

Eighteen of the HIV-infected (81.8%) and 47 (60.3%) of the uninfected patients had active HBV infection (HBsAg seropositive) (*p* = 0.06). The two patients who were HIV infected and had no evidence of HBV infection, both had histological confirmation of the diagnosis. One of the two HIV-infected patients had a history of alcoholic liver disease as a risk factor.

HBV genotypes were determined for 43 cases, nine of whom were HBV/HIV co-infected. All nine of the co-infected patients and 25/34 (74%) of the mono-infected patients had HBV genotype A. HCC cases infected with HBV genotype A had a median age of 36 years (IQR: 31–48) compared to 53 years (IQR: 50–58) in those infected with HBV genotype D, *p* = 0.008.

### Prevalence of HCV infection

Antibodies to HCV were detected in 10 of 101 cases (9.9, 95% CI: 4.1–15.7). There was insufficient sample for testing in six cases. The mean age of the anti-HCV positive subjects was 57.5 years (95% CI: 45.8–69.2), while that of the anti-HCV negative cases was 12 years younger, 44.6 years (95% CI: 41.8–48.0) *p* = 0.01. Of the ten anti-HCV seropositive HCC cases, 9 (90%) were male. Among the ten anti-HCV positive cases, three were HBV/HCV co-infected while one case had triple infection with HCV, HBV and HIV. Of eight anti-HCV positive cases, 5 (63%) had detectable HCV RNA with a median of 6.27 log10 copies/ml (IQR: 5.67 log10–6.42 log10). Samples from four HCV-infected HCC cases were successfully sequenced - two cases were infected with HCV genotype 5a while one case each had genotype 1a and the other genotype 1b.

### Survival of HCC patients

**Fig. 1:**
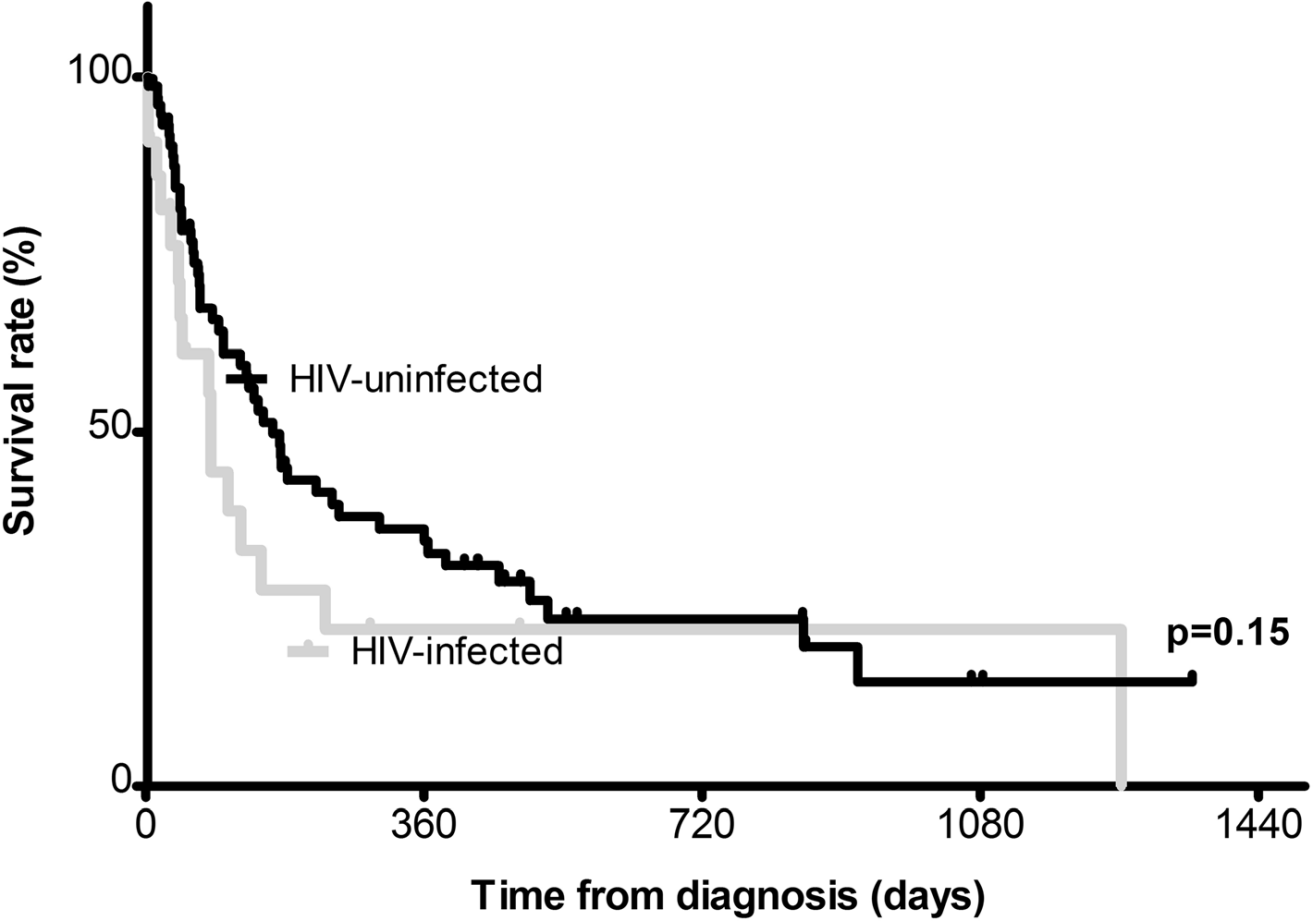
Survival rates according to known HIV status. Comparison between Kaplan–Meier survival curves in 22 HIV-infected patients with HCC and 78 HIV-uninfected HCC patients. Patients whose HIV status was unknown were not included in this survival analysis

Of the 22 HIV-infected HCC patients, 15 (68.2%) died during follow up, while of the 78 patients without HIV, 44 (56.4%) died during follow-up. The Kaplan-Meier survival curves of the two groups were not significantly different, *p* = 0.15. The survival curves of the HCC cases with and without HIV infection are shown in Fig. 1. Median survival was 82 days among the HIV-infected patients and 181 days among those without HIV.

## Discussion

Primary liver cancer accounts for 600,000 deaths each year [24]. HCC is the most common primary liver cancer, accounting for over 90% of all cases [25]. Vaccination has been shown to reduce the incidence of HBV-related liver cancer. However, the hepatitis B vaccine was not introduced into South Africa’s Expanded Programme on Immunisation until 1996. Prior to that time, childhood-acquired HBV infections were common in much of Africa. All the HCC patients recruited into this study were born before 1996.

The prevalence of HIV among HCC patients in this study was 22% (95% CI: 14–30), higher than the prevalence of HIV in the South African adult population, estimated at 14.4% among females and 9.9% among males from the 2012 South African National HIV Prevalence, Incidence and Behaviour Survey Report [26]. Moreover, the prevalence was particularly high among females in this study; 36% of whom were HIV-infected. The majority of HIV/HBV co-infected women were African Blacks, evidence of the vulnerability of this group to HIV infection within sub-Saharan Africa [27, 28]. HIV/HBV co-infected females were about 10 years younger at HCC diagnosis than HBV mono-infected females, although the difference did not reach statistical significance. Our observations are consistent with those of a few African studies that have evaluated the effect of HIV on the epidemiology of HCC in Africa [13]. These studies have reported that HIV-infected liver cancer patients were 10 years younger at diagnosis than patients without HIV.

The patients with HBV infection were younger at HCC diagnosis than those that were infected with HCV in this study. This finding is consistent with reports that HCV-associated HCC tends to peak in the sixth decade of life and HBV-associated HCC in the fourth decade [29]. The differences in age at presentation probably reflect the ages of acquisition of viral hepatitis infection and the differing oncogenic processes of these two viruses. In sub-Saharan Africa, HBV infection is predominantly acquired early in childhood through vertical and horizontal transmission, while HCV is acquired, predominantly in high-risk groups, in adulthood. HCV associated HCC is more often associated with cirrhosis than HBV associated HCC, and therefore presenting later in life either in the context of liver cirrhosis.

We found that patients who were infected with HBV genotype A presented at a significantly younger age than those with genotype D. That finding is consistent with that of Kew et al., who described a mean age difference of almost 7 years [30]. The frequent absence of HBeAg and the basal core promotor mutations in genotype D compared to genotype A may provide an explanation for the difference in age at presentation between the two genotypes. HBeAg positivity, which normally denotes high HBV replication, is a known risk factor for HCC development and tends to be more prevalent, together with the basal core promoter mutations, in patients infected with genotype A compared to those with D [31].

Fifty-five percent of HCC patients in this study were Black Africans who are thought to be at higher risk of HCC than other racial groups due to greater exposure to factors predisposing to HCC, such as dietary iron overload and HBV and HIV infection [32, 33]. Cirrhosis prevalence in HBV-associated HCC among African patients has previously been reported to be 44–63% [34–36]. However, only 23% of our patients had known underlying cirrhosis at HCC diagnosis, probably because their diagnoses of cirrhosis were based on clinical rather than histological parameters. Clinical assessment of cirrhosis is known to result in under-diagnosis and under-reporting of its true prevalence, especially among patients with compensated cirrhosis. Studies from the United States have also found that cirrhosis was not identified among 24–30% of HCC patients who had underlying cirrhosis [37, 38]. In patients with hepatitis C, HCC invariably develops following cirrhosis such that early diagnosis of malignancy is diminished when cirrhosis is diagnosed late [38]. However, cirrhosis is not necessary for the development of HCC in CHB.

Our findings raise questions regarding implementation of current hepatitis B treatment and HCC screening guidelines in South Africa. Patients with viral hepatitis have access to tertiary level treatment and surveillance programmes but without wider access to screening for HBV infection, it is not possible to implement them. Unfortunately, many HBV infections are only diagnosed once they present with HCC. Few of our HBV-mono-infected patients had been screened and treated for CHB before their HCC diagnosis. As a result, many patients have radiological findings of multiple or large liver lesions at presentation. Only 30% of cases in this current study had histologic diagnosis of HCC. This has implications with regard to understanding the true burden of HCC with South Africa whose national cancer registry is pathology-based. As a result, there is an underestimation of the burden of HCC and this impacts on the allocation of resources towards the health burden.

Among these HCC patients in this study, survival was dismal regardless of HBV or HIV status. Although the HIV-infected HCC patients had a median survival of 82 days, compared to 181 days among those without HIV infection, the difference was not statistically significant. Nonetheless, the poor survival of HIV-infected patients compared to HIV-uninfected patients argues against the probability that the difference in age at diagnosis between HIV-infected and uninfected HCC patients is due to lead-time bias. More intense surveillance of HIV-infected than uninfected patients would be expected to lead to earlier HCC diagnosis with better prognosis and survival which was not observed in this study.

An important limitation of this study is recruitment bias; because we recruited HCC cases only from oncology units of state-owned tertiary hospitals, we may have missed patients presenting with HCC at primary health care facilities and therefore unable to recruit all cases. In the setting of a strong health care system, such patients might be diagnosed earlier and survive longer. However, anecdotal evidence suggests that even if patients presented to the primary care facilities with early-stage HCC, those facilities are not equipped or staffed with appropriately trained individuals to provide early detection and effective treatment. It is more likely that our recruitment approach missed patients with HCC who did not survive long enough (after referral to confirm a tentative diagnosis) to be seen at the recruiting sites for this study. It is unlikely that HIV and HBV infection would be less prevalent among such patients than among those in this cohort.

Our findings suggest that HIV infection may cause early development of HCC in HBV-infected patients, particularly in females, and that many patients who present late with HCC have underlying undiagnosed and/or untreated HBV infection. Estimates suggest that only 5% of patients infected with viral hepatitis are aware of their infection status prior to HCC diagnosis [39]. Most of the HIV-infected patients in our study were aware of their HIV status. However, very few were aware of their HBV status.

## Conclusions

Our results highlight the need to address the problem of hepatitis B within sub-Saharan Africa. The majority of childhood-acquired HBV infections become chronic, and CHB is the greatest risk factor for HCC in sub-Saharan Africa. HBV birth dose vaccination is the cornerstone to the HBV prevention strategy and should be available to all infants, particularly those born in high prevalence countries. The South African Ministry of Health takes the position that it is unnecessary to screen HIV-infected individuals for HBV because the current first line ART that includes tenofovir and lamivudine is active against both HIV and HBV. However, the hypothesis that ART is sufficient to prevent the evolution of HCC in HIV-infected patients needs further study. In order to identify those most at risk of HCC, there is a need to screen HIV-infected individuals for HBV, to treat those who test positive for HBV, and to vaccinate those who are not immune. In addition, better screening and management strategies for HCC should be developed for sub-Saharan African populations, where young people, especially those infected with HIV are at risk of this rapidly fatal malignancy.

## Data Availability

The data that support the findings of this study are available from Stellenbosch University upon reasonable request and with the permission of Stellenbosch University.

## Abbreviations

HIV: human immunodeficiency virus
HBV: hepatitis B virus
HCC: hepatocellular carcinoma
HCV: hepatitis C virus
CHB: chronic hepatitis B
HBsAg: HBV surface antigen
ART: antiretroviral therapy
SSA: Sub-Saharan Africa
AFP: alpha fetoprotein
95% CI: 95% confidence interval
